# Multivariate selection drives concordant patterns of pre- and postcopulatory sexual selection in a livebearing fish

**DOI:** 10.1038/ncomms9291

**Published:** 2015-09-15

**Authors:** Alessandro Devigili, Jonathan P. Evans, Andrea Di Nisio, Andrea Pilastro

**Affiliations:** 1 Department of Biology, University of Padova, via Ugo Bassi 58/b, 35131 Padua, Italy; 2 Centre for Evolutionary Biology, School of Animal Biology, University of Western Australia, 35 Stirling Highway, Western Australia, 6009 Crawley, Australia

## Abstract

In many species, females mate with multiple partners, meaning that sexual selection on male traits operates across a spectrum that encompasses the competition for mates (that is, before mating) and fertilizations (after mating). Despite being inextricably linked, pre- and postcopulatory sexual selection are typically studied independently, and we know almost nothing about how sexual selection operates across this divide. Here we bridge this knowledge gap using the livebearing fish *Poecilia reticulata*. We show that both selective episodes, as well as their covariance, explain a significant component of variance in male reproductive fitness. Moreover, linear and nonlinear selection simultaneously act on pre- and postcopulatory traits, and interact to generate multiple phenotypes with similar fitness.

## Abstract (web summary)

In species in which females mate with multiple partners, sexual selection acts on male traits involved in mating and fertilization. Here, the authors show that selection acting before and after mating explains a significant component of variance in male reproductive fitness in a livebearing fish.

## Graphical abstract

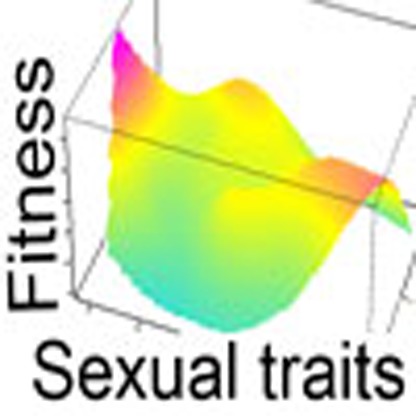

It is well established that in many species sexual selection rarely ends at mating, and that sperm from rival males typically compete for fertilization (sperm competition)^1^ or are subject to female-moderated processes that bias fertilization towards particular males (cryptic female choice)^2^. Consequently, a male's net reproductive fitness during a given reproductive episode will depend both on his ability to secure mates (precopulatory sexual selection) *and* on how successful his sperm are at fertilizing eggs in the presence of ejaculates from rival males (postcopulatory sexual selection). A key question, however, surrounds the relative importance of these pre- and postcopulatory episodes of selection in determining male reproductive fitness^3^,^4^.

The realization that polyandry (female multiple mating) is taxonomically widespread^5^, and therefore a key arbiter of sexual selection in most species^6^, has generated enormous empirical interest in sperm competition and cryptic female choice over the past four decades^7^,^8^. However, it is only recently that researchers have questioned the extent to which both pre- and postcopulatory sexual selection contribute towards (male) reproductive fitness. In particular, a recent series of studies has adopted quantitative statistical approaches to partition variance in reproductive fitness into components attributable to mating (precopulatory) success and siring (postcopulatory) success^9^,^10^,^11^,^12^. Collectively, these studies are important because they quantitatively verify, and sometimes challenge, previously held assumptions that polyandry (female multiple mating) generates intense postcopulatory sexual selection on male (and female) traits.

In addition to accounting for the relative importance of pre- and postcopulatory selection in determining success in siring offspring, we need to know what aspects of the phenotype contribute to variance in male reproductive success (MRS). To our knowledge, there are currently no studies characterizing multivariate selection on postcopulatory traits within a sexually selected framework (but see refs 13, 14 for examples of multivariate selection on ejaculate traits within a natural selection framework). Yet, the net action of selection on male reproductive traits will depend critically on how pre- and postcopulatory episodes of sexual selection interact to determine male reproductive fitness (see also ref. 15). Thus, to gain a more complete understanding of how selection operates during pre- and postcopulatory episodes of sexual selection, we require studies that both decompose net reproductive fitness into its relative constituent (pre- and postcopulatory) variance components^9^,^10^,^11^,^12^ *and* characterize the direction, strength and form of multivariate selection on male traits across successive episodes of sexual selection.

In the present study, we combine analyses of phenotypic selection with variance component decomposition to gain insights into pre- and postcopulatory sexual selection in the guppy, *Poecilia reticulata*. Guppies are livebearing, highly polyandrous freshwater fish used as models for studying pre- and postcopulatory sexual selection^16^,^17^. In guppies, precopulatory sexual selection is driven primarily by female choice^16^. However, guppies are also highly polyandrous, and therefore sperm from multiple males typically compete to fertilize eggs^17^. Prior research on guppies has established that a male's success in sperm competition is positively associated with courtship behaviour^18^ and body colour^19^,^20^. This covariance is thought to be due to a combination of factors, including the positive association between precopulatory traits (courtship and colour ornamentation) and ejaculate quality^21^,^22^,^23^ and females exerting ‘cryptic' preferences for sperm from attractive males^24^. What remains to be established, however, is an understanding of the relative importance of pre- and postcopulatory processes in determining male reproductive fitness. In this paper we address this question by evaluating the relative contributions of male mating success (MMS) and postcopulatory success (PCS) in explaining male reproductive fitness. We set up replicate populations of freely interacting males and females and use molecular paternity assignment to reconstruct the mating success and siring success of each male within our replicate populations. We then quantify the relative contribution of these two components of fitness in explaining overall MRS. Finally, to complement these analyses we use phenotypic data collected from each male across all populations to estimate the form, strength and direction of multivariate linear and nonlinear sexual selection on pre- and postcopulatory traits.

We find that pre- and postcopulatory success, as well as their covariation, contributes equally to explain the variance in male reproductive fitness. This result indicates that postmating sexual selection is particularly strong in this species^25^. Furthermore, using multivariate selection analysis we reveal complex fitness surfaces and concordant patterns of selection on pre- and postcopulatory male traits, which contribute explaining the observed covariation between pre- and PCS.

## Results

### Paternity analysis and male reproductive fitness

**Figure 1:**
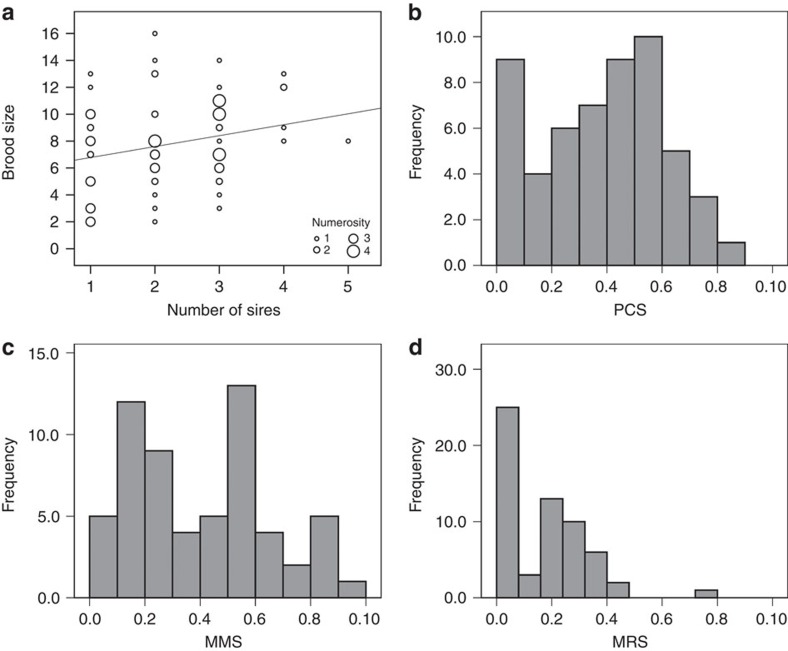
Descriptive statistics of data from replicated populations. (**a**) Correlation (Pearson's *r*=0.246, *P*=0.044, *n*=68) between number of sires per brood and brood size, where different sizes for circles correspond to different number of cases. Frequency distributions of male (**b**) postcopulatory success (PCS, *n*=54), (**c**) mating success (MMS, *n*=60) and (**d**) reproductive success (MRS, *n*=60), respectively.

We quantified MRS from 10 replicate groups, each of which contained six males and eight females that were housed together for 7 days (see Methods). MRS was calculated as the proportion of offspring sired by each male (*n*=60) over the total offspring produced in his replicate group. Seventy-two of the eighty females used in the experiment produced broods (*n*=532; mean brood size±s.d.=7.39±3.59; range=1–16) from which 530 offspring could be assigned unambiguously to one sire (99.6%). Four females produced a single offspring and therefore multiple paternity could not be assessed in these broods. Our microsatellite paternity analyses confirmed that 47 females (∼69%) produced offspring sired by two or more males (mean±s.d. sires=2.18±1.01; range 1–5). Because female guppies are able to store sperm for several months, some females (*n*=54) produced second broods, which we also genotyped for parentage analysis (see Methods). Although we do not consider these second broods for estimating MRS (see below), they were helpful in identifying females that had demonstrably mated with more males than those that were identified only through paternity analysis of the first brood (see Methods). The analysis of paternity in these second broods revealed an additional four females that had mated with two or more males during the mating trials (that is, females producing offspring in the first brood that were sired by just one male but who subsequently produced at least one offspring from a different male). With the inclusion of these data in our analysis, our revised estimate of female mating rate increased to 2.40±1.1 s.d. mates per female (range 1–5). The number of sires per brood was positively correlated with brood size (Pearson's *r*=0.246, *P*=0.044, *n*=68; Fig. 1a).

The mean male PCS, corrected for the number of males competing in a single brood (see Methods), was 0.38±0.24 s.d. (range=0.00–0.81, Fig. 1b). Males sired offspring with an average of 2.53±1.94 s.d. females (range 0–7) but mated with an average of 2.88±1.85 s.d. females (range 0–7). Since the number of females that produced a brood varied across tanks (mean±s.d.=7.2±0.79, range=6–8), estimates of MMS were expressed as the number of females with which the male mated over the total number of females that produced offspring (mean±s.d.=0.40±0.26, range=0–1.00, Fig. 1c). The mean proportion of offspring sired by males (MRS) was 0.17±0.15 s.d. (range=0–0.75, Fig. 1d).

### Partition of MRS variance into its MMS and PCS components

Our variance-partitioning analysis revealed that ∼40% of the variance in MRS was explained by MMS, 38% by PCS and 41% by the covariance between MMS and PCS (Table 1). As pointed out in ref. 26, the sum of these components can exceed 100% because the total variance is not simply the sum of its component variances and covariances but higher-order terms (the products of variances and covariances) and skewness in the data also contribute to the total. MMS and PCS were positively correlated (Pearson's *r*=0.581, *n*=54), indicating that males that do well in obtaining mates also do well when competing for fertilization. The observed correlation coefficient was significantly higher than the expected (simulated) correlation coefficient due to the estimation of MMS from paternity data (mean simulated *r*=0.108, *P*=0.0001, Monte Carlo simulation based on 10,000 replicates; see Methods), indicating that the observed covariation between MMS and PCS is greater than expected by chance.

### Multivariate selection analysis and fitness surfaces

**Figure 2:**
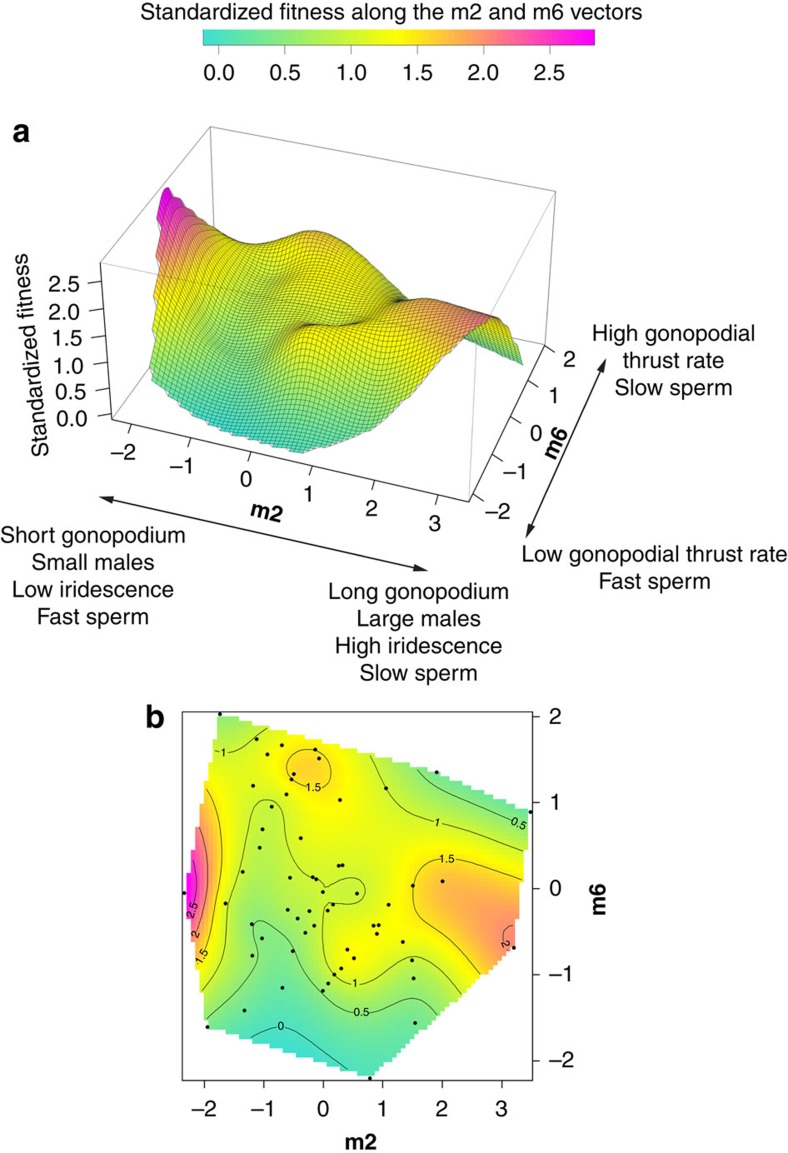
Fitness surface based on m2–m6 vectors. Three-dimensional (**a**) and contours (**b**) fitness surfaces. The vectors **m2** and **m6** represent the strongest axes disruptive and stabilizing selection; **m2** is positively loaded by gonopodium length and iridescent and body area and negatively by sperm velocity; **m6** is loaded positively by gonopodial thrust rate and negatively by sperm velocity. Standardized fitness is shown.

**Figure 3:**
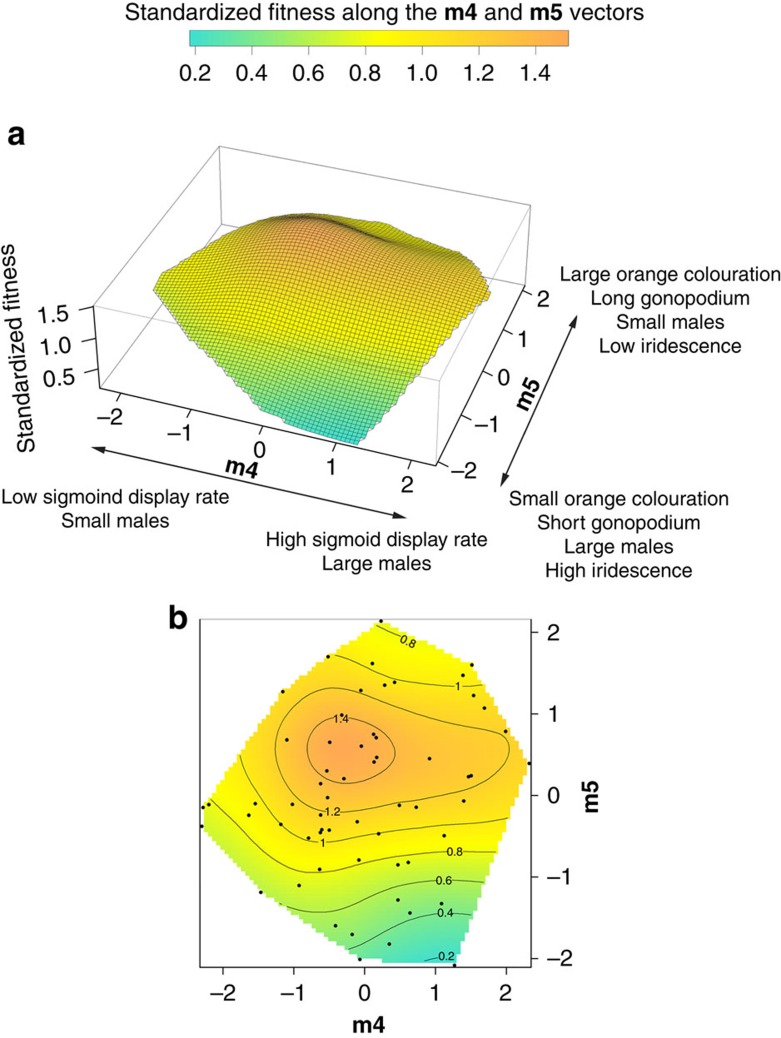
Fitness surface based on m4–m5 vectors. Three-dimensional (**a**) and contours (**b**) fitness surfaces. The **m4** vector is mainly positively loaded by display behaviour (and partially by body area), while **m5** is loaded positively by orange colouration (and weakly by gonopodium length, body area and iridescent area). Standardized fitness is shown.

We detected significant positive linear (*β*) selection on gonopodium length (Table 2) and significant negative nonlinear selection (*γ*) on iridescent area and gonopodial thrust frequency (see *γ* coefficients on diagonal in Table 2). We also found evidence for positive correlational selection on gonopodium length and iridescent colouration (see Table 2). We conducted canonical rotation of the *γ* matrix, which generates a matrix of new composite trait scores (eigenvectors, **m1, m2, …m7**, in which trait representation is similar to that of a principal component analysis), each describing a major axis of selection in the fitness surface^27^,^28^. Following this, we detected nonlinear selection on four **m** vectors, revealing significant disruptive (**m2**) and stabilizing selection (**m4**, **m5** and **m6**; see Table 3 and Figs 2 and 3). The **m2** vector was primarily loaded by gonopodium length (+) and secondarily by body size (+), sperm velocity (−) and iridescent area (+). The **m4** vector yielded a negative eigenvalue and was primarily loaded by courtship display rate (+) and body size (+), while the (negative) **m5** vector was loaded by orange colouration (+), gonopodium length (+), body size (−) and iridescent colouration (−). Finally, **m6**, with the highest negative significant eigenvalue, was strongly associated with gonopodial thrust rate (+) and sperm velocity (−). Fitness surfaces were obtained by fitting thin-plate splines on the significant major axes of selection (**m2**, **m4**, **m5** and **m6**). We illustrate the strongest pattern of disruptive selection with vectors **m2**–**m6** (Fig. 2) and stabilizing selection with vectors **m4**–**m5** (Fig. 3). Other possible combinations (that is **m2**–**m4**, **m2**–**m5** and so on) yielded little further information (see Supplementary Figs 1–4).

## Discussion

Our study yields critical insights into how successive episodes of sexual selection interact to determine male reproductive fitness in guppies. The results of our quantitative analyses indicate that both pre- and postcopulatory sexual selection are important determinants of male reproductive fitness. Moreover, our analyses reveal that the covariance between pre- and postcopulatory sexual selection explains a large proportion of the variance in MRS. Our study therefore reinforces previous evidence from Trinidadian guppies that paternity success following consecutive double mating favours phenotypically attractive males^18^,^19^. However, we extend these previous analyses by exploring how selection acts during successive episodes of sexual selection. Specifically, by generating estimates of relative fitness for each of the males participating in this study, we were able to explore the form, strength and direction of selection on pre- and postcopulatory traits. The evidence from these latter analyses points to complex patterns of nonlinear selection in which multiple phenotypes enjoy similar fitness. These patterns of nonlinear selection reveal correlational selection on combinations of traits involved in pre- and postcopulatory sexual selection and patterns of disruptive and stabilizing selection along major axes of multivariate selection.

Recent studies that have assessed the relative importance of pre- and postcopulatory episodes of sexual selection have yielded mixed results. Pischedda and Rice^10^, for example, used stepwise regression of lifetime (male) reproductive success against mating success and fertilization success in *Drosophila melanogaster*. They showed that, after controlling for mating rank, only a small fraction (1.9%) of MRS could be attributed to variation in fertilization success. By contrast, postcopulatory sexual selection constituted far stronger sources of selection in two other recent studies, contributing 46% of the variance in reproductive success in the red jungle fowl *Gallus gallus*^9^ and 36% in the freshwater snail *Physa acuta*^11^. Our experiment similarly confirms that postcopulatory sexual selection contributes a significant source of variance to overall reproductive fitness, but also, as found in the jungle fowl^29^, that the positive covariance between pre- and postcopulatory components of selection are equally important in explaining variance in MRS.

Our design precluded the opportunity of directly observing all copulations by focal males, and thus some males that achieved zero paternity but still mated may have been overlooked in our estimates of PCS. In this way, we may yet have underestimated the importance of postcopulatory processes in this system. Indeed, Collet *et al.*^29^ recently showed how estimates of MMS based exclusively on behavioural observation *or* genetic parentage analysis can yield misleading results. However, our estimate of female multiple mating rate (average of 2.4 males per female) is similar to that found in a similar (unpublished) study in which all copulations among replicate groups of six males and six females were observed over the course of five days (average of 2.53 mates per female, number of replicates=5; Cattelan, S, Morbiato, E. & Pilastro, A, unpublished data). Thus, although our estimates of MMS are derived mainly from paternity data rather than direct observations of copulations, our ensuing estimates of the relative importance of pre- and postcopulatory sexual selection in determining MRS are likely to be realistic.

In addition to revealing evidence for directional selection on gonopodium length, which may be attributable to female preferences for longer gonopodia^30^ and/or greater insemination success by such males^31^, our analyses reveal correlational selection on gonopodium length and iridescent colouration. However, other potentially correlated traits are not considered in the analyses of correlation gradients. The canonical rotation of the *γ* matrix addresses this problem by generating vectors (described by trait combinations) that represent the axes where selection is most intense^27^,^28^. When we applied this method, we found four significant major axes of nonlinear selection that were consistent with patterns of stabilizing and disruptive selection on several traits, including gonopodium length and iridescence. Specifically, we found evidence for disruptive selection on **m2** (positively loaded by gonopodium length, body and iridescent area and negatively loaded by sperm velocity) and stabilizing selection on **m6** (loaded primarily by gonopodial thrust rate and again sperm velocity; see Fig. 2). Thus, selection favours (*i*) large males with high iridescence, long gonopodia and low sperm velocity, or (*ii*) the reverse pattern. This pattern of selection corroborates previous evidence for a negative genetic correlation (that is, trade-off) between sperm velocity and iridescent area in guppies^32^. Furthermore, we found evidence for two smaller peaks close to the centre of **m2** and extreme values of **m6** (Fig. 2). These peaks may, to some extent, be explained by the relatively strong loadings of sperm velocity on both axes of selection, thus emphasizing how interactions among traits can generate relatively complex fitness surfaces and consequently multiple phenotypes with similar fitness^33^. The second major fitness surface (Fig. 3) revealed a single largely intermediate peak for **m4** and **m5**, which is consistent with stabilizing selection on body area, courtship displays and orange pigmentation. However, it should also be noted that the ridge in the fitness surface corresponding to above average values of **m4** (strongly positively loaded by sigmoid display rate) indicates that males performing higher than average courtship display rates were still favoured by selection. Again, this finding corroborates previous work on guppies revealing paternity skews in favour of males that perform high courtship rates^18^.

In conclusion, our analyses reveal concordant patterns of pre- and postcopulatory sexual selection in guppies. However, our selection analyses also suggest that there is unlikely to be a single high-fitness phenotype that maximizes MRS in guppies, a finding that mirrors prior evidence for multiple fitness peaks in this species in the context of precopulatory components (attractiveness) of selection^33^. To the best of our knowledge, the present study is the first to combine analyses of phenotypic selection with variance partitioning methods to dissect and understand the net action of pre- and postcopulatory sexual selection. Our ensuing results emphasize the limitations of focusing on individual components and/or episodes of sexual selection, which may erroneously support the notion of unrelenting selection favouring extreme trait evolution.

## Methods

### Study population and maintenance

We used sexually mature guppies descended from wild-caught fish collected from the Lower Tacarigua River in Trinidad. Guppies were reared in mixed-sex groups (∼1:1 sex ratio) and routinely rotated among large (130 l, 0.70 fish l^−1^) tanks to maintain an outbred stock population (12:12 light/dark cycle at 26 °C). All fish were fed a mixed diet of commercial dry flake food and *Artemia* nauplii. Female fish used in this experiment were virgin (that is, reared in female-only groups) to ensure that they were initially sexually receptive and that matings could be attributed exclusively to focal males. A summary of the reproductive parameters of males and females is presented on Table 4. We used a reasonable number of individuals to provide sufficient statistical power.

This experiment was conducted according to the Italian legal requirements and was approved by the Ethics committee of the University of Padova (permit no. 36/2011 to A.P.).

### Experimental design

We set up *n*=10 replicate populations of guppies, each comprising six males (aged 3–4 months) and eight virgin females (4–5 months). Before placing each group into their allotted mating tanks (65 l, 55 × 35 × 40 cm), males and females were lightly anaesthetized (with a 150-mg l^−1^ solution of tricaine methanesulfonate, MS222), photographed for subsequent analysis of colour patterns (males, see below) and measured for body size (both sexes: standard length (SL)=distance in mm between the snout and tip of caudal peduncle, and body area). We ensured that male and female SL did not significantly differ among the 10 groups (males: *F*_9,50_=0.42, *P*=0.92; females: *F*_9,66_=1.16, *P*=0.34). After assigning males and females to their respective tanks, the groups were allowed to interact freely for 7 days, during which we recorded male mating behaviour on three successive occasions (see below). After 7 days, males were removed from the mating tanks and isolated individually for 5 (±1) days in tanks (2 l) placed adjacent to two tanks, each containing one female. Providing males with visual (but not direct) access to females ensured that they would have remained sexually motivated during the recovery phase and had sufficient time to replenish their sperm stores before the assessment of ejaculate traits (see below). For their part, females were removed from the mating tanks after 7 days and isolated individually until they produced their first and second broods. Because female guppies store sperm for several months, any offspring produced in the second brood would have resulted from fertilizations from stored sperm. Tissue samples were then collected from all fish (putative sires, females and offspring) for paternity assignment using microsatellite markers (see below).

### Male mating behaviour

We observed the mating behaviour of all males in each tank during three successive observation periods. Males were individually recognizable from their unique colour patterns, which were sketched before the first observation. As virgin female guppies are initially sexually receptive to male courtship when they first encounter males^16^, we scored copulation success for each of the six males shortly after (5 min) males were placed in the tank. This initial observation period (day 1) lasted for 1 h, during which two observers simultaneously observed the six males (three males per observer) to estimate the number of successful matings and courtship displays (where males arch their body in an s-shaped ‘sigmoid' posture and quiver to display their colour patterns to the female). We then carried out two subsequent behavioural observations on days 2 and 5 of the experiment. On both days, we counted the number of successful matings, courtship displays (hereafter ‘sigmoids') and forced copulation attempts (‘gonopodial thrusts'), the latter of which are characterized by males attempting to forcibly inseminate females without prior courtship. On both days, observations lasted 10 min and males were observed in a haphazard order to avoid order effects. All behaviours were standardized for observation time (that is, number of behaviours per minute). No copulations were observed on days 2 and 5.

### Male morphology and sperm velocity

We measured the body area and the area of colour spots, including orange (total area of carotenoid and pteridine spots) and iridescence (total area of blue, green, silver and violet structural colour patches), from the digital photographs of each male. The length of the male's gonopodium (intromittent organ) was estimated from these images by measuring the distance (within 0.01 mm) from the base of the gonopodium to its distal tip. Sperm velocity was estimated using computer-assisted sperm analyses (CASA), as described previously^34^. Briefly, sperm were stripped from anaesthetized males by applying gentle pressure to the male's abdomen^35^. Sperm bundles (spermatozeugmata) were collected using a sequencing pipette (model 203, volume 0.05–3 μl, Drummond Scientific Company) and immediately used for CASA (CEROS, Hamilton-Thorne). Sperm velocity assays were performed twice for each male using three spermatozeugmata per sample after activation with 150 mM KCl mixed with 4 mg l^−1^ bovine serum albumin. CASA sperm velocity assays give significantly repeatable measures^21^,^36^. As reported previously for guppies^21^,^37^, the three resultant velocity estimates (VAP: average path velocity; VSL: straight line velocity; and VCL: curvilinear velocity) were highly correlated (*r*≥0.72); we therefore collapsed these measures to a single principal component score (SPC1) that explained 92.5% of the variation in our sample and predominantly described sperm velocity (that is, strongly positively loaded by VAP, VSL and VCL).

### Paternity analysis

Genomic DNA was extracted from the whole-body tissue of offspring and from the caudal fin of adults using a Chelex protocol^20^,^38^. The tissue samples obtained from 72 mothers, the putative fathers (*n*=60) and all of the offspring (*n*=532) were collected and stored in a freezer at −80 °C until used for analysis. We used five microsatellite loci to assign paternity to males within each of the 10 populations (see Table 5). PCR amplifications were performed using the GeneAmp PCR System 9700 Thermocycler (Applied Biosystems, CA, USA). The PCRs were performed with 7.64 μl BDH, 1 μl MgCl_2_, 3 μl Taq buffer, 0.53 μl dNTPs, 0.38 μl primers (forward+reverse), 0.08 μl Taq DNA polymerase (Promega) and 2 μl DNA template. The cycling protocol included an initial denaturation step at 95 °C for 1 min, 30 cycles of 10 s denaturation at 95 °C, 30 s annealing (T listed in Table 5), 30 s extension at 72 °C and a final extension for 5 min at 72 °C. Amplified fragments were separated by electrophoresis on an ABI PRIMS DNA Analyzer 3100/3700 sequencer (ABI PRISM, Applied Biosystems), using 400 HD ROX (Perkin-Elmer, Applied Biosystems) as a size standard. PCR products were visualized using the Peak Scanner software ( www.appliedbiosystems.com) and paternity was assigned to offspring using CERVUS 3.0 (refs 39, 40).

### Measures of MRS

We estimated MRS for each male (*n*=60) by calculating the proportion of offspring sired by the male over all the offspring produced in his replicate group. Our estimates of MRS are therefore comparable among replicates, where the number of offspring produced by females was variable. We estimated MMS from the paternity data by calculating the number of females with which each male produced at least one offspring^41^. Since not all females produced broods (*n*=8 of 80 females did not produce offspring), we adjusted MMS within each replicate group by expressing each male's mating success (number of females with whom he produced at least one offspring) as a proportion of the total number of females that produced offspring in the tank. As with other studies that rely on paternity data to estimate mating rates, this MMS estimate potentially underestimates actual mating success because some males that successfully mate with a female may fail to sire offspring. To offset this problem, we incorporated two additional sources of data to refine our estimates of MMS. First, we used paternity data obtained from each of the females that produced a second brood (*n*=54 broods comprising 473 offspring). From these data we were able to identify cases in which males sired offspring in the second brood but not the first, clearly indicating that the males had successfully copulated during the mating trials. Second, we used behavioural data obtained during the mating trials to identify males that copulated successfully but nevertheless failed to sire offspring in either of the broods (additional six cases detected).

We estimated male PCS as the mean proportion of offspring sired by males that unambiguously mated with at least one of the females in the population. As we note above, whether or not a male was assigned a PCS score depended on paternity data from the first and second broods as well as behavioural observations confirming successful copulation. Our estimates of PCS, however, were derived only from the first broods produced by each female. Six of the 60 males were neither observed mating, nor obtained paternity in either of the two broods (that is, their MMS was 0) and hence were not assigned a PCS estimate (final sample size for PCS was therefore 54). In generating the PCS estimates, we took the precaution of only considering broods in which there was the unambiguous potential for sperm competition to occur (that is, only those coming from multiply mated females, including those females subsequently shown to mate multiply using paternity data from the second broods—see above). In generating the PCS estimates, we adjusted each male's score to take account of the number of males that sired at least one offspring within a brood. In this way we standardized each males' PCS to account for the *realized* number of sperm competitors within each brood. Thus, a male that achieves a mean paternity share of 50% in a brood comprising just two sperm competitors is given the same adjusted mean PCS score as a male that sires 33% of offspring against two rivals, 25% against three rivals and so on. We used the following formula to obtain adjusted PCS scores:


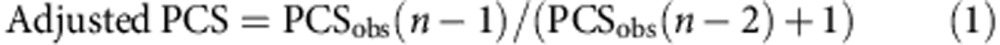


Where PCS_obs_ is the observed proportion of offspring sired by a focal male in the brood, and *n* is number of males competing for fertilization.

### Simulated correlation between PCS and MMS

The use of paternity data to estimate both MMS and PCS may potentially generate a spurious positive correlation between pre- and postcopulatory sexual selection. To illustrate the problem, consider a situation in which all males have equal MMS but vary in the competitiveness of their ejaculates and therefore exhibit variance in PCS. Deriving MMS estimates from paternity data could potentially lead to higher estimates of mating success by highly successful sperm competitors simply because of the increased likelihood of detecting offspring from males with highly competitive sperm across multiple females (that is, the most successful sperm competitors are likely to successfully sire offspring among more females than their less competitive counterparts). Thus, despite no actual variance in MMS, the ability to detect sire alleles in resultant broods would be influenced by PCS, thus generating a positive correlation between MMS and PCS. To address this issue, we estimated the extent to which the methods used to estimate MMS and PCS have the potential to generate such spurious relationships. To this end, we used a simulated paternity data set in which the expected MMS and PCS (hence MMS_exp_ and PCS_exp_) values for each male varied independently one from the other. On the basis of the simulated MMS and PCS scores we generated simulated paternity data to calculate the a posteriori MMS and PCS estimates (that is, derived from the resulting paternity distribution, hence MMS_sim_ and PCS_sim_) and hence the correlation coefficient between MMS_sim_ and PCS_sim_. Using a Monte Carlo procedure, this simulation was repeated 10,000 times and the distribution of the correlation coefficients between MMS_sim_ and PCS_sim_ (*r*_sim_) was compared with the correlation coefficient (*r*_obs_) between the observed MMS and PCS (hence MMS_obs_ and PCS_obs_). All simulations (see also below) were done using PopTools 3.2.5 (ref. 42) and are available upon request to the authors. In particular, for each simulation we assigned each male a PCS_exp_ value, which was drawn from an empirical PCS distribution with mean 0.50 (s.d.=0.209, range=0.053–0.947, *n*=68)^38^. In our simulation, each male could potentially mate with several females. We assumed (conservatively) that the expected within-male PCS did not vary across females. In each replicated simulation, the probability that each male would have mated with one (or more) randomly chosen female(s) was drawn from a Poisson distribution with a mean equal to the MMS observed in our original data set (0.40). The two components of MRS (MMS and PCS) were therefore independent in our simulation. As expected, preliminary simulations revealed that if the MMS_obs_ (0.40) value was used in the simulation, the resulting paternity-based MMS_sim_ was slightly lower than the MMS_obs_, because some of the matings did not result in paternity (mean MMS_sim_=0.353±0.024 s.e.). Through successive approximations we increased the MMS_exp_ used in the simulation (0.474) until the resulting average, paternity-based MMS_sim_ (0.40) was equal to MMS_obs_. The distribution of simulated correlation coefficients between MMS_sim_ and PCS_sim_ was compared with the observed value correlation coefficient and *P* value was calculated as the proportion of simulations in which the simulated correlation was larger than the observed value. In particular, for each simulation we assigned each male a PCS_exp_ value, which was drawn from an empirical PCS distribution with mean 0.50 (s.d.=0.209, range=0.053–0.947, *n*=68)^38^. In our simulation, each male could potentially mate with several females. We assumed (conservatively) that the expected within-male PCS did not vary across females. In each replicated simulation, the probability that each male would have mated with one (or more) randomly chosen female(s) was drawn from a Poisson distribution with a mean equal to the MMS observed in our original data set (0.40). The two components of MRS (MMS and PCS) were therefore independent in our simulation. As expected, preliminary simulations revealed that if the MMS_obs_ (0.40) value was used in the simulation, the resulting paternity-based MMS_sim_ was slightly lower than the MMS_obs_ because some of the matings did not result in paternity (mean MMS_sim_=0.353±0.024 SE). Through successive approximations we increased the MMS_exp_ used in the simulation (0.474) until the resulting average, paternity-based MMS_sim_ (0.40) was equal to MMS_obs_. The distribution of simulated correlation coefficients between MMS_sim_ and PCS_sim_ was compared with the observed value correlation coefficient and *P* value was calculated as the proportion of simulations in which the simulated correlation was larger than the observed value.

### Variance in male pre- and PCS

We followed previously described variance decomposition methods^26^ to partition variance in MRS among effects attributable to MMS, PCS and their covariance ([cov(MMS, PCS)]. In this way, our model accounts for fitness components that simultaneously (through associations/trade-offs) contribute towards MRS (see ref. 26 and appendix in ref. 43). The following model partitions total variance in MRS into two fitness components plus their associated covariance^26^:


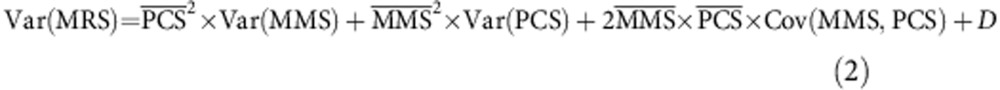


where *Var* and *Cov* are variances and covariances, respectively, for the fitness components defined previously (MRS, MMS and PCS), and *D* is the difference between the summed variances and covariance and the computed value of *Var*(MRS)^26^. 95% Confidence intervals for the three variance component estimates were obtained from the bootstrap distribution based on 10,000 samples with replacement using PopTools 3.2.5 (ref. 42; Table 1).

### Multivariate selection analysis

We used selection analyses and response surface methodology to characterize the form, intensity and direction of multivariate selection on pre- and postcopulatory sexual traits. All 60 males were used in this analysis. All phenotypic traits were standardized to have the mean of zero and s.d. of one. To estimate relative fitness of each male, we followed ref. 44 by standardizing the MRS values used in the variance partition analysis above to a mean of one (divided by observed population mean). Linear selection gradients (*β*), which describe directional selection, and the matrix of nonlinear (quadratic and correlational) selection gradients (*γ*), were estimated using a multiple regression approach^44^ on the following seven traits: (*i*) frequency of sigmoid displays; (*ii*) frequency of gonopodial thrusts (both standardized to rates per minute); (*iii*) male body area; (*iv*) gonopodium length; (*v*) orange spot area; (*vi*) iridescent spot area; (*vii*) sperm velocity (SPC1). Quadratic regression coefficients were doubled to obtain estimates of stabilizing/disruptive (nonlinear) selection gradients^45^. We estimated *β* separately with a common linear regression and subsequently estimated *γ* gradients with a full quadratic regression^44^. We conducted a canonical rotation of the gamma matrix to test for nonlinear selection on trait combinations^37^,^38^. This method generates a matrix of new composite trait scores (eigenvectors, **m1, m2, …m7**, in which trait representation is similar to that of a principal component analysis), each describing a major axis of selection in the fitness surface^27^,^28^. The strength of nonlinear selection (curvature of the surface) along each eigenvector was estimated from its eigenvalue (*λ*), while the form of selection was deduced by its sign (positive indicative of disruptive selection; negative indicative of stabilizing selection). This methodology simplifies the interpretation of the gamma matrix by reducing the number of variables and removing the effect of correlational selection on paired traits. As pointed out in ref. 27, this method does not add information to the regression analysis but simplifies its interpretation. To avoid type I errors, which are commonly associated with the double regression method^46^, we used the methodology proposed in ref. 47 based on a standard permutation procedure (no. of permutations=10,000), using R script (version 3.0.2, http://www.r-project.org/) provided by the authors. To visualize fitness surfaces we fitted thin-plate splines using the Tps function in the ‘fields' package of R. This nonparametric approach provides a less constrained view of fitness surfaces than the best quadratic approximation^33^. Nonparametric surface visualization is a key instrument to interpret results from selection analyses as regression coefficients describe surface curvature and not directly selection type.

## Additional information

**How to cite this article:** Devigili, A. *et al.* Multivariate selection drives concordant patterns of pre- and postcopulatory sexual selection in a livebearing fish. *Nat. Commun.* 6:8291 doi: 10.1038/ncomms9291 (2015).

## Supplementary Material

Supplementary Information Supplementary Figures 1-4

## Figures and Tables

**Table 1:** Variance in male pre- and postcopulatory success.

	**MRS**	**MMS**	**PCS**	**Covariance**	***D***
Unstandardized	0.023	0.009	0.009	0.010	−0.004
Standardized	0.842	0.341	0.316	0.347	−0.162
% Explained	100	40.5	37.5	41.3	−19.2
95% CI		26.0–64.6	24.8–57.3	27.6–59.3	−68.3–12.7

CI, confidence interval; MMS, male mating success; MRS, male reproductive success; PCS, postcopulatory success.

Variance in MRS explained by variance in MMS, *PCS* and their covariance (*n*=60) following ref. 26. *D* represents the remainder term accounting for high-order terms (the products of variances and covariances) and skewness in the data^26^. 95% CIs were obtained from a bootstrap distribution (10,000 samples with replacement).

**Table 2:** Linear and nonlinear selection gradients.

**Traits**	***γ*** **coefficients**						
	**Gonopodium**	**Body area**	**Iridescence**	**Orange**	**Sperm velocity**	**Sigmoid displays**	**Gonopodial thrusts**
Gonopodium	0.352 (0.531)	0.040 (0.869)	**0.944** (**0.001**)	−0.105 (0.605)	−0.248 (0.206)	0.011 (0.961)	0.242 (0.360)
Body area		0.288 (0.569)	−0.103 (0.825)	0.711 (0.068)	−0.167 (0.505)	−0.361 (0.129)	0.190 (0.451)
Iridescence			−**1.826** (**0.045**)	−0.519 (0.128)	−0.371 (0.206)	0.155 (0.409)	−0.149 (0.623)
Orange				−0.912 (0.137)	−0.387 (0.237)	−0.275 (0.128)	−0.167 (0.524)
Sperm velocity					−0.794 (0.100)	−0.045 (0.857)	0.498 (0.148)
Sigmoid displays						−0.322 (0.478)	−0.044 (0.846)
Gonopodial thrusts							**−1.486** (**0.001**)
*β* coefficients	**0.292** (**0.033**)	−0.096 (0.533)	−0.144 (0.359)	−0.026 (0.851)	−0.007 (0.959)	0.003 (0.985)	0.168 (0.230)

*β* and *γ* coefficients obtained with multiple regressions. In the diagonal quadratic selection coefficients are shown and represent disruptive (+) or stabilizing (−) selection acting on trait. Above the diagonal correlational selection coefficients represent traits selected to be positively (+) or negatively (−) correlated. In parenthesis *P* values obtained by a multiple regression model in which male reproductive success (*n*=60) was the dependent variable and the standardized male traits and their quadratic terms were the independent variables^44^.

In bold significant coefficients.

**Table 3:** Matrix of eigenvectors.

**Eigenvector**	**Eigenvalue λ**	**Gonopodium**	**Body area**	**Iridescence**	**Orange**	**Sperm velocity**	**Sigmoid displays**	**Gonopodial thrusts**
**m1**	0.988 (0.583)	0.452	−0.562	0.365	−0.469	−0.009	0.356	0.018
**m2**	**0.758** (**0.046**)	0.659	0.476	0.315	0.209	−0.419	−0.144	−0.009
**m3**	−0.009 (0.979)	−0.339	−0.164	0.067	0.193	−0.690	0.363	−0.457
**m4**	**−0.438** (**0.019**)	−0.049	0.501	−0.002	−0.024	0.192	0.817	0.204
**m5**	**−0.848** (**0.002**)	0.371	−0.386	−0.364	0.709	0.146	0.228	0.069
**m6**	**−1.292** (**0.010**)	−0.114	−0.126	−0.182	−0.072	−0.507	−0.018	0.822
**m7**	−1.691 (0.291)	0.305	0.125	−0.773	−0.436	−0.185	0.036	−0.261

Matrix of eigenvectors representing the major axes of nonlinear selection. Nonlinearity of selection (curvature of the surface) is given by its eigenvalue (*λ*), and the shape by its sign (positive-disruptive, negative-stabilizing selection). Each trait has a loading factor that can be interpreted similarly to those of a principal component analysis. In parenthesis *P* values obtained by permutation methodology (no. of permutations=10,000) proposed by Reynolds *et al.*^47^.

In bold significant eigenvalues.

**Table 4:** Descriptive statistics (means) of the reproductive parameters of females and males in each tank.

	**Tank number**										
	**1**	**2**	**3**	**4**	**5**	**6**	**7**	**8**	**9**	**10**	**Mean**±**s.d.**
*Females*											
Females with brood	7	6	8	7	7	8	8	6	7	8	7.20±0.79
Brood size	5.71	6.17	6.87	8.57	7.2	6.75	8	7.5	8.71	8.12	7.36±1.00
Time to parturition (days)	27.43	31.86	36.00	26.71	34.57	30.75	32.50	36.17	44.14	38.13	33.82±5.19
Sires per brood*	1.43	2.20	3.13	3.17	2.43	1.86	1.88	2.5	1.43	1.86	2.19±0.62
Singly sired broods*	4	1	0	1	2	3	3	0	4	3	2.10±1.52
Offspring number	6.67	6.17	9.17	10.00	8.17	9.00	10.67	7.50	10.17	10.83	8.83±1.66
*Males*											
Male mating success (no. of females)	2.17	2.50	4.33	3.83	3.00	2.67	2.83	3.00	1.83	2.67	2.88±0.74
Male mating success (proportion of females)	0.31	0.42	0.54	0.55	0.43	0.33	0.35	0.50	0.26	0.33	0.40±0.10
Standardized postcopulatory success	0.44	0.45	0.43	0.41	0.36	0.46	0.41	0.50	0.41	0.44	0.43±0.04

^*^Broods with a single offspring were excluded from this analysis.

**Table 5:** Microsatellite loci used to estimate paternity in the experiment.

**Microsatellite locus**	**Bp range**	**No. of alleles**	**GenBank accession no.**	***T***_**a**_ **(°C)**	**Reference**
Kond15	244–296	14	AF368429	52	48
TTA	102–163	15	AF164205	52	49
PR 80	142–168	10	AF467905	54	50
PR 40	244–298	11	AF467904	56	50
Agat11	240–371	21	BV097141	56	51

Bp, base pairs length; *T*_a_, annealing temperature.
