# Supplementary material for: Multivariate selection drives concordant patterns of pre- and postcopulatory sexual selection in a livebearing fish
Source: Nat Commun. 2015 Sep 15;6:8291. doi: 10.1038/ncomms9291 (PMC4579849; doi:10.1038/ncomms9291)
Supplement: Supplementary Information — Supplementary Figures 1-4 [file ncomms9291-s1.pdf]

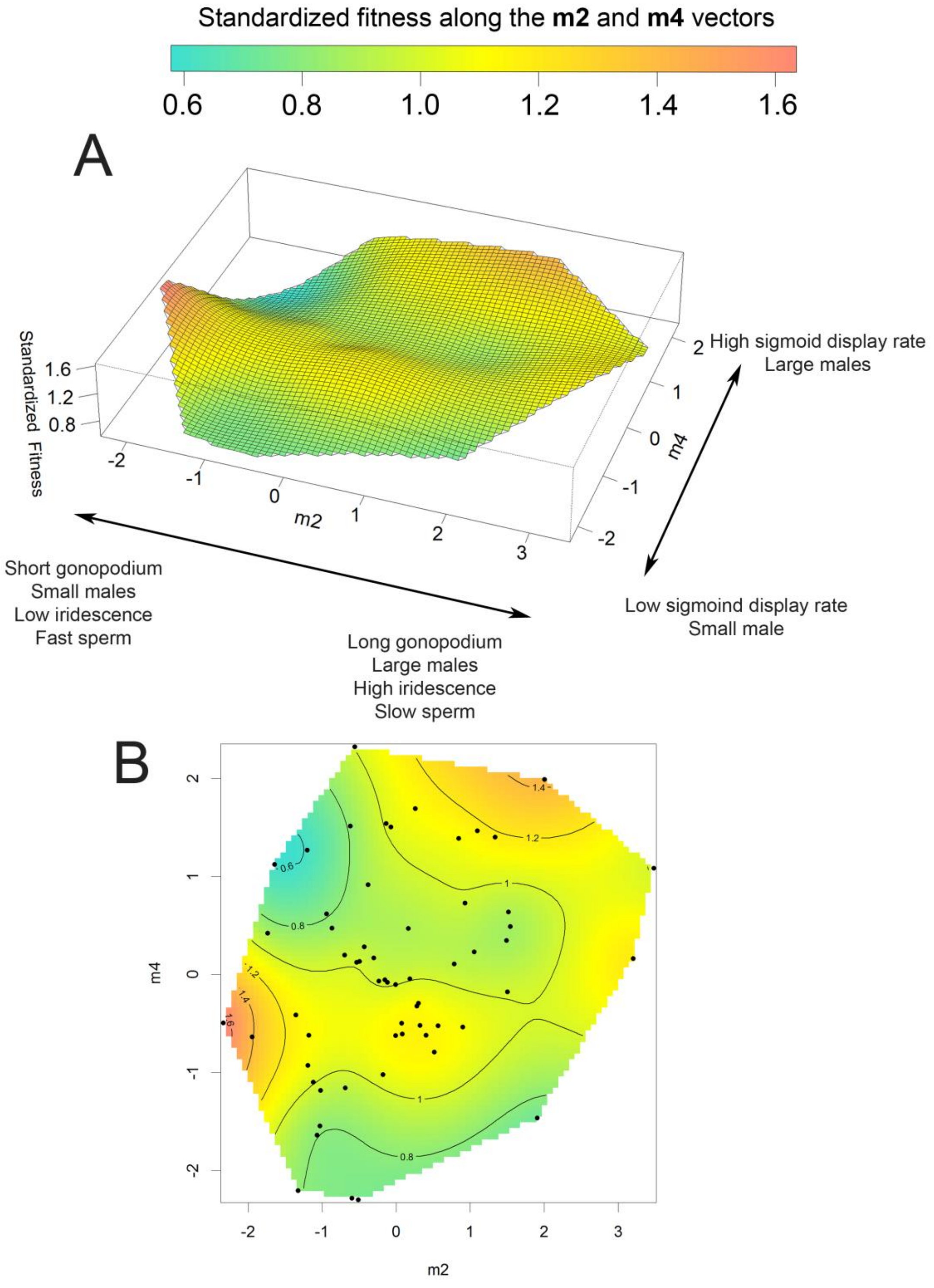

Supplementary Figure 1**.** Fitness surface based on **m2-m4** vectors

Three-dimensional (A) and contours (B) fitness surfaces. The **m2** vector is positively loaded by gonopodium length and iridescent and body area and negatively by sperm velocity; **m4** vector is mainly positively loaded by display behaviour (and partially by body area). Standardized fitness is shown.

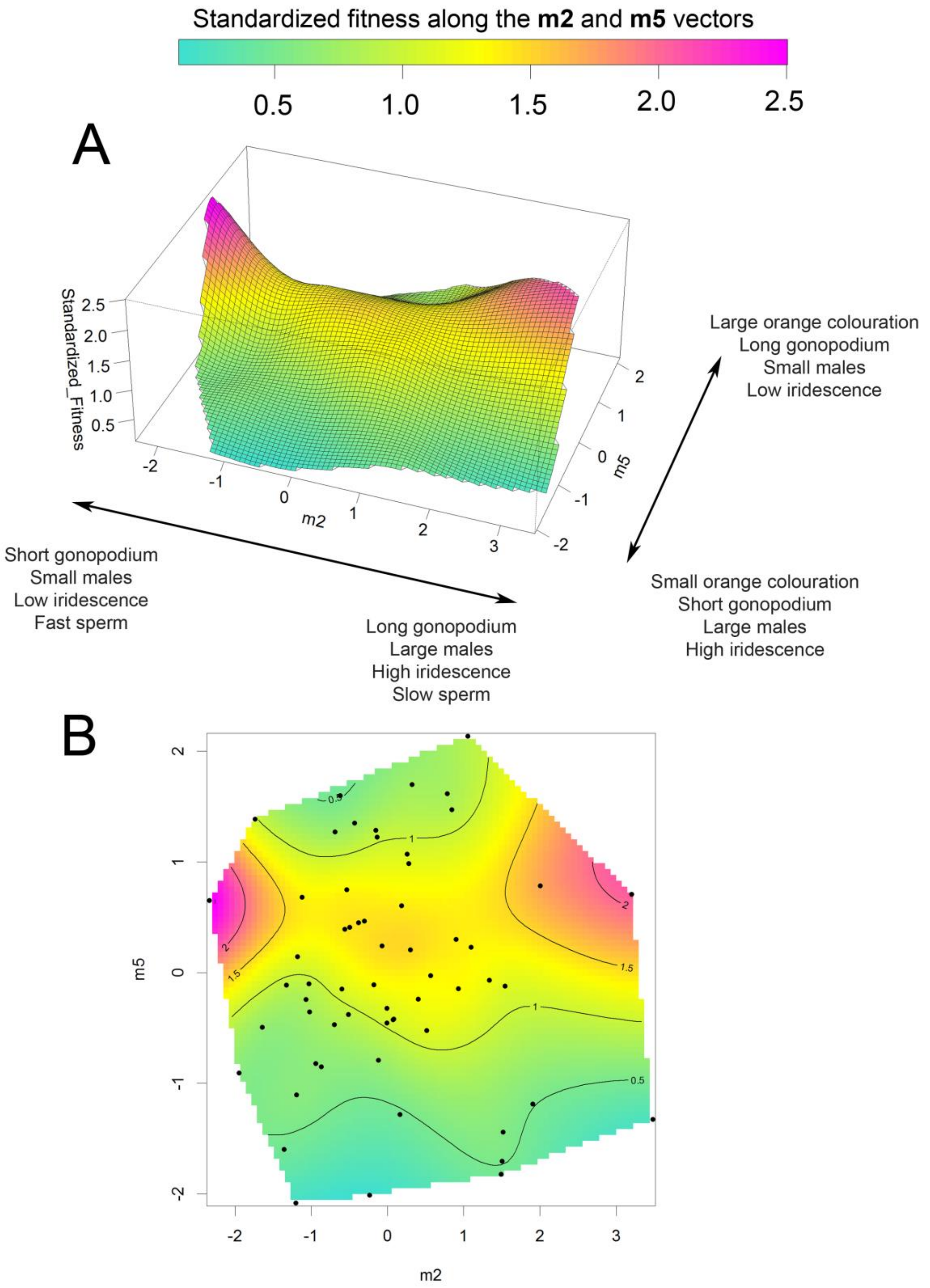

Supplementary Figure 2. Fitness surface based on **m2-m5** vectors

Three-dimensional (A) and contours (B) fitness surfaces. The **m2** vector is positively loaded by gonopodium length and iridescent and body area and negatively by sperm velocity; **m5** is loaded positively by orange coloration (and weakly by gonopodium length, body area and iridescent area). Standardized fitness is shown.

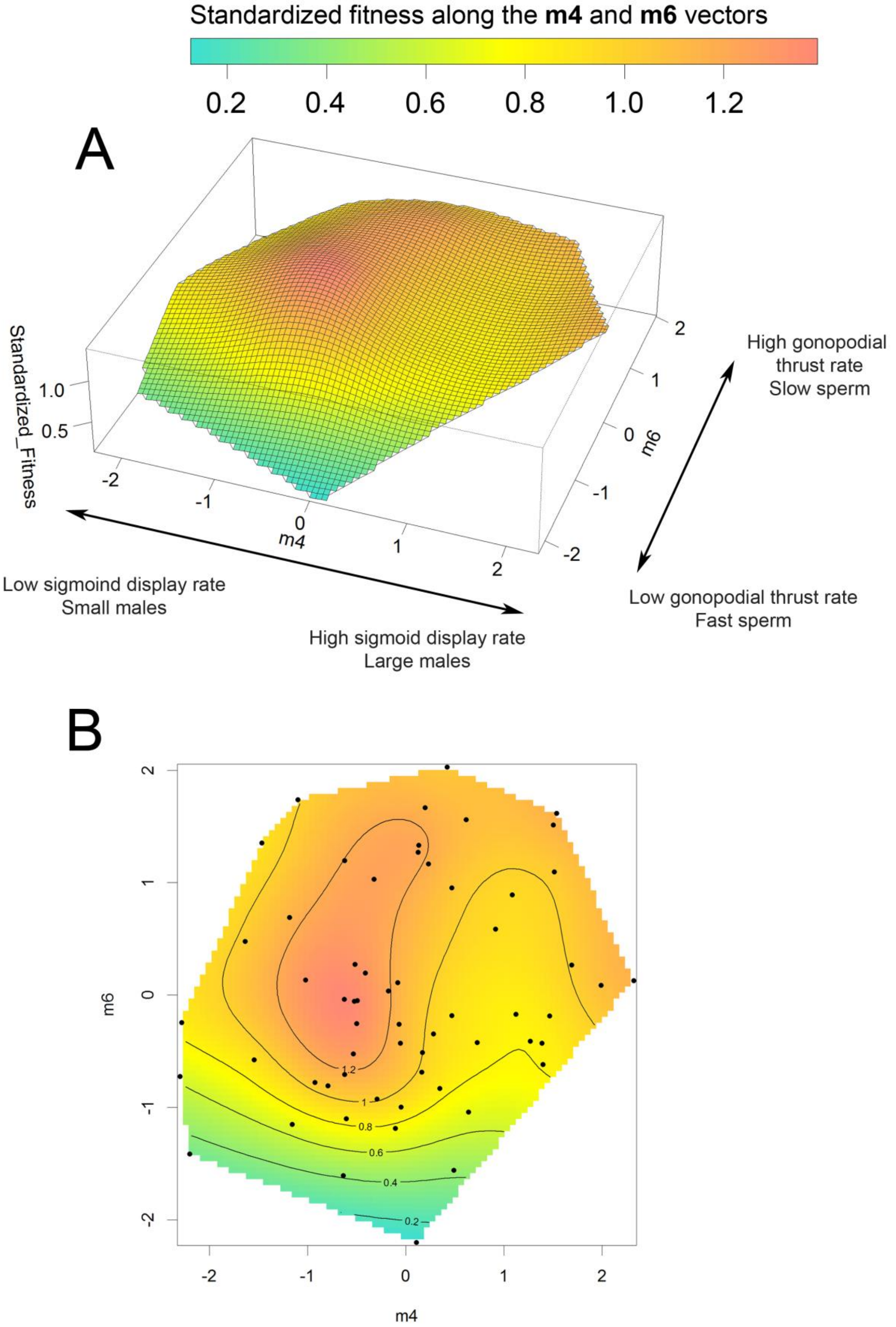

Supplementary Figure 3. Fitness surface based on **m4-m6** vectors

Three-dimensional (A) and contours (B) fitness surfaces. The **m4** vector is mainly positively loaded by display behaviour (and partially by body area) while **m6** is loaded positively by gonopodial thrust rate and negatively by sperm velocity. Standardized fitness is shown.

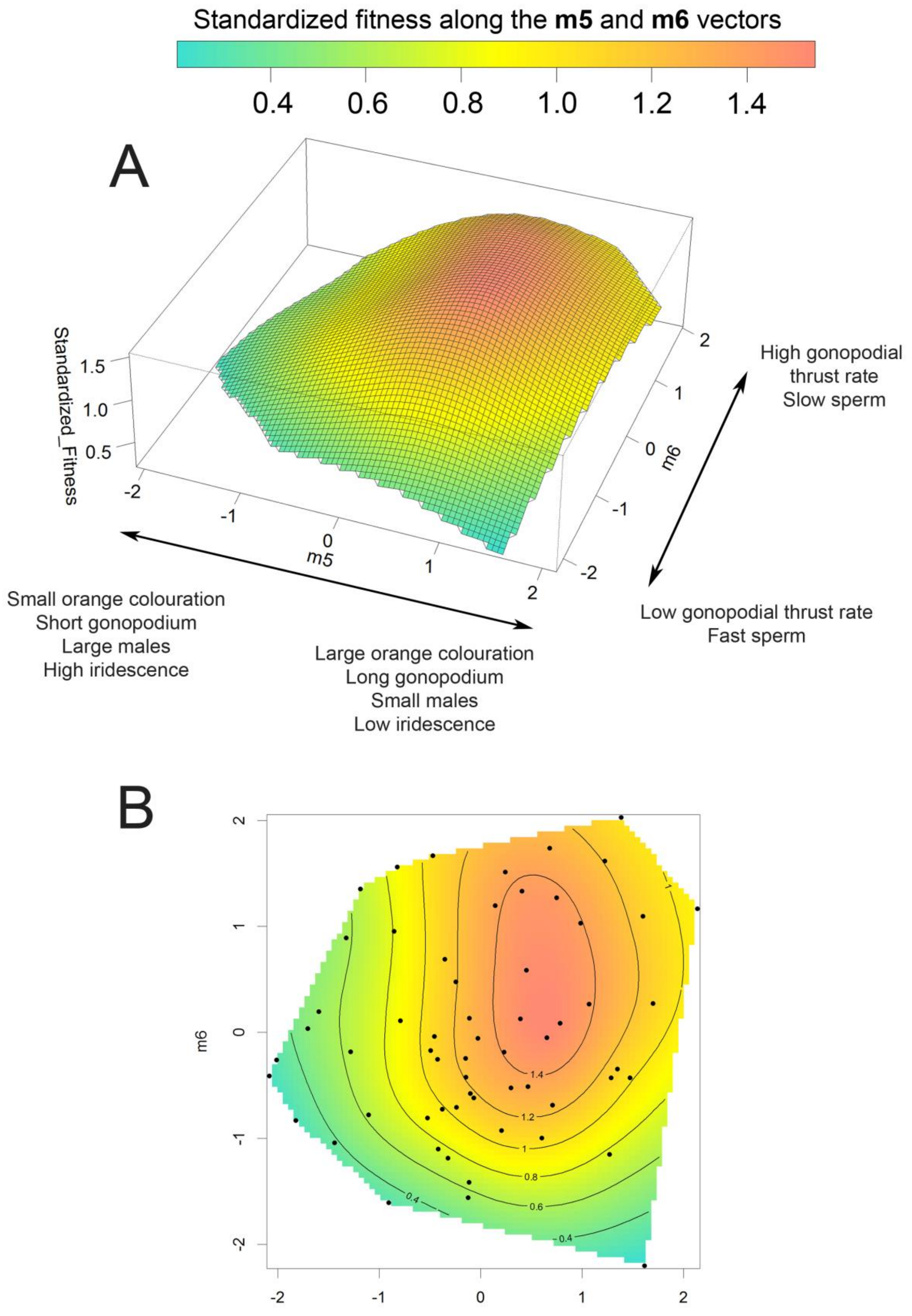

Supplementary Figure 4. Fitness surface based on **m5-m6** vectors

Three-dimensional (A) and contours (B) fitness surfaces. The **m5** vector is loaded positively by orange coloration (and weakly by gonopodium length, body area and iridescent area) while **m6** is loaded positively by gonopodial thrust rate and negatively by sperm velocity. Standardized fitness is shown.
